# Development of a sustainable portable Archimedes screw turbine for hydropower generation

**DOI:** 10.1038/s41598-025-90634-8

**Published:** 2025-02-18

**Authors:** Nouman Khan, Muhammad Kaleem Sarwar, Muhammad Rashid, Hafiz Kamran Jalil Abbasi, Saif Haider, Muhammad Atiq Ur Rehman Tariq, Abdullah Nadeem, Muhammad Ahmad Zulfiqar, Ali Salem, Nadhir Al-Ansari, Abdelaziz M. Okasha, Ahmed Z. Dewidar, Mohamed A. Mattar

**Affiliations:** 1 Centre of Excellence in Water Resources Engineering, University of Engineering and Technology, Lahore, 54890 Pakistan; 2 College of Engineering, IT and Environment, Charles Darwin University, NT 0810, Darwin, Australia; 3 College of Engineering and Science, Victoria University, Melbourne, 8001 Australia; 4 Department of Mechanical Engineering, University of Engineering and Technology, Lahore, 54890 Pakistan; 5 Civil Engineering Department, Faculty of Engineering, Minia University, Minia 61111, Egypt; 6 Department of Civil, Environmental, and Natural Resources Engineering, Lulea University of Technology, 97187 Luleå, Sweden; 7 Department of Agricultural Engineering, Faculty of Agriculture, Kafrelsheikh University, Kafr El-Sheikh 33516, Egypt; 8 Prince Sultan Bin Abdulaziz International Prize for Water Chair, Prince Sultan Institute for Environmental, Water and Desert Research, King Saud University, P.O. Box 2454 Riyadh 11451, Saudi Arabia; 9 Department of Agricultural Engineering, College of Food and Agriculture Sciences, King Saud University, P.O. Box 2460, Riyadh 11451, Saudi Arabia; 10 Structural Diagnostics and Analysis Research Group, Faculty of Engineering and Information Technology, University of Pécs, Pécs 7622, Hungary

**Keywords:** Archimedes screw turbine, Physical model, Design parameters, Angle of inclination, Hydraulic efficiency, Civil engineering, Mechanical engineering

## Abstract

Portable hydropower turbines are turbines with a scale below 5 kW and which can be carried from one place to another easily by hand due to their light weight. This study was carried out to evaluate the potential of Archimedes Screw Turbine (AST) as an improved portable hydro-power turbine (PHPT) to address shortcomings in available portable turbines. The design of Archimedes screw hydro-power turbine is mainly concerned with screw geometry, which is determined by a variety of internal and external characteristics, including its length, external and internal diameter, Pitch of blades, and Number of the blades, which were 80 cm, 18 cm, 9.53 cm, 18 cm and two number of blades respectively. The turbine was manufactured from stainless steel material according to design parameters and installed in the laboratory. Experimental testing was performed at different discharges (Q) of 0.3, 0.4, 0.5, 0.6, and 0.7 ft^3^/s and at the angle of inclination of 22, 30, 45, and 55° of screw shaft to measure power outputs and overall efficiencies. The maximum overall efficiency obtained was 70% at a flow rate of 0.5 ft^3^/s and at an angle of inclination of 30°. The power output at maximum overall efficiency was 42 watts and hydraulic efficiency was 75.5%. At the flow rate of 0.3 ft^3^/s and an angle of inclination of 55°, the turbine produced a minimum power output of 22.8 watts and an overall efficiency of 39.4%.Experimentation revealed that the flow rate (Q) and inclination of the turbine shaft affect the turbine Power output (P_o_) and overall efficiency (η_o_). This study helps to manufacture small AST on a large scale, to utilize small flows of water, and to evaluate the possibilities of AST as an appropriate portable hydro-power generation turbine. Further research and experimentation are needed to assess whether 3D printing can be effectively scaled for broader implementation in low-resource areas.

## Introduction

Electricity is essential for productivity in every field of life, yet many remote or temporary sites lack stable power due to their distance from the grid^1^. Unreliable power access, particularly in developing countries, also leads to substantial economic losses, with electricity outages alone contributing to these setbacks^2^. Global infrastructure projects face significant cost increases due to power access limitations, with cost overruns averaging 66.3% per project, totaling $388 billion across 401 projects^3^. Diesel generators remain a common fallback; however, they are responsible for high emissions, emitting approximately 2.68 kg of CO₂ per liter of diesel consumed and contributing significantly to project carbon footprints^4^. Furthermore, logistical issues around fuel transport to isolated areas add up to $250,000 annually in costs for mid-sized projects, making diesel use both economically and environmentally unsustainable^5^. In river-adjacent sites, renewable energy solutions backed by available resources offer an innovative alternative to diesel, capable of generating consistent power from flowing water^6^. Therefore, unreliable power access in remote construction sites drives significant cost overruns and environmental impact, emphasizing portable renewable energy solutions, particularly in river-adjacent areas, as a sustainable alternative for consistent power generation.

The turbines are generally categorized into two main types: impulse and reaction turbines. Impulse turbines, such as Pelton and Turgo turbines, convert the kinetic energy of water into mechanical energy using high-velocity jets^7^. Reaction turbines, such as Francis and Kaplan turbines, harness both the pressure and kinetic energy of water to generate torque and are often used in low to medium-head hydroelectric facilities^8,9^. A third category includes quasi-static turbines like the Archimedes screw turbine (AST), which operates by converting the weight of water into mechanical energy in low-head, low-flow conditions^10^. ASTs are particularly effective in generating electricity with minimal water head and variable flow rates, making them ideal for micro-hydropower applications in remote areas^11^. They offer financial benefits by reducing capital expenditures compared to traditional turbines like Kaplan turbines^12^, and their simple design leads to lower maintenance costs, making them suitable for off-grid locations^13^. Additionally, ASTs are known for being fish-friendly, which minimizes environmental impacts typically associated with hydropower installations, such as damage to migratory species^14^. However, despite their advantages, the lack of standardized design criteria for ASTs and their relatively new application presents challenges, particularly in terms of optimization and scaling^15^.

The development of a portable power source is driven by the increasing need for reliable, decentralized energy solutions in remote and off-grid locations, where access to traditional power infrastructure is limited or unavailable^16^. In such areas, where conventional energy sources are either impractical or too costly, micro-hydropower systems, particularly the Archimedes screw turbine (AST), offer a promising solution. These turbines can efficiently generate power from small streams, irrigation channels, and wastewater flows, even in low-head, low-flow conditions, without the need for complex installations or large-scale infrastructure^17^. This makes the AST an ideal choice for locations where traditional hydropower is not feasible due to geographical or infrastructural limitations^18^. Furthermore, the portable design of the Archimedes screw turbine allows for easy deployment in various settings, such as construction sites and rural areas, where access to electricity is critical. In these contexts, fossil fuel-powered generators are often the default energy source, yet they are both costly to operate and harmful to the environment, contributing to high operational costs and increased emissions^13,19^. The Archimedes screw turbine offers a cleaner, more cost-effective, and sustainable alternative, addressing the energy needs of remote sites. However, the design and development of AST is a complex task that needs thorough consideration, particularly in the context of portability.

Analytical approaches have been established for the design of Archimedes screws. However, certain design elements of Archimedes screw remain to depend on experimental knowledge^20^. The absence of analytical guidelines in the design of the Archimedes screw turbine (AST) is a significant drawback, often cited as a major disadvantage^11^. The designs are now depending upon the designer’s expertise^21^. Prominent research in non-English design literature on ASTs includes^15,21–25^. The major English studies were conducted by^26^ and^15^, But as^11^ pointed out, none of these techniques are simple to comprehend and put into practice, especially when it comes to preliminary estimates and the early phases of AST hydropower project design.

The Archimedes Screw Turbine (AST) consists of key components including the screw shaft, housing (web), drive shaft, and bearings. These components work together to convert water flow into rotational motion, enabling energy generation. A chain drive system facilitates the connection between the AST shaft and the electric generator (electric dynamo). This system incorporates two gears shown in Fig. 7: a primary (larger) gear attached to the turbine shaft and a secondary (smaller) gear connected to the electric generator (or dynamo) through the chain. The gear ratio is 1:12, meaning the generator rotates 12 times for every rotation of the turbine shaft.

The advantages of open AST are their light weight, easy to transport, and efficient in low-head, low-flow conditions, including their straightforward helical screw design simplifies manufacturing makes them cost-effective and easy to maintain. The disadvantages of open AST prone to debris buildup, corrosion, and weathering. Their power output is lower compared to enclosed AST, and they may not perform well in turbulent water conditions.

Moreover, ASTs are known for their low maintenance requirements, with major maintenance typically needed only after 20 to 30 years of operation. This makes them highly suitable for remote or off-grid installations where regular maintenance may be challenging. Due to their simple design, AST can be manufactured locally and can be easily repaired using basic tools available in the field, such as wrenches, hammers, screwdrivers, and welding kits. This ease of construction, maintenance and repair is particularly beneficial for rural or isolated areas, minimizing downtime and reducing overall maintenance costs.

The northern areas of Pakistan are rich in huge hydropower resources^27^. The recoverable potential of micro-hydropower in Gilgit-Baltistan is estimated to be 300 MW from perennial streams. This potential needs to be tapered through PHPT units for the isolated population of GB province^28^.

A number of investigations examined the total efficiency and power production of the Archimedes screw turbine^29^ at varying flow rates, heads, and angles of inclination of the screw shaft. Maulana et al.^30^ examined a two-blade Archimedes screw turbine with an inner radius of 0.077 m, an outer radius of 0.1435 m, and a length of 2 m, capable of functioning under low-flow situations with heads below 10 m. The flow discharge rates of 0.025 m^3^/s, 0.0125 m^3^/s, and 0.0044 m^3^/s were analyzed. The maximum turbine efficiency is 55% at a flow rate of 0.0125 m^3^/s, with peak rotation and power at 295 rpm and 116.10 Watts, respectively. Abdullah et al.^29^ examined the effect of turbine slope (β) at angles of 22, 30, and 40° on power output across different hydraulic heads. The study data indicate that a maximum efficiency of 49% produces 1.4 W of power. This efficiency is attained by running the turbine at a 22-degree inclination. CFD analyses were conducted^31^ to evaluate the impact of blade angle on the efficiency of a spiral horizontal axis hydro turbine. The turbine screw shaft angle was adjusted to 15°, 18°, 21°, and 30° at water inlet velocities of 1 m/s, 1.5 m/s, and 2 m/s. The results of the research indicate that screw inclination angle significantly influences turbine efficiency.

**Fig. 1:**
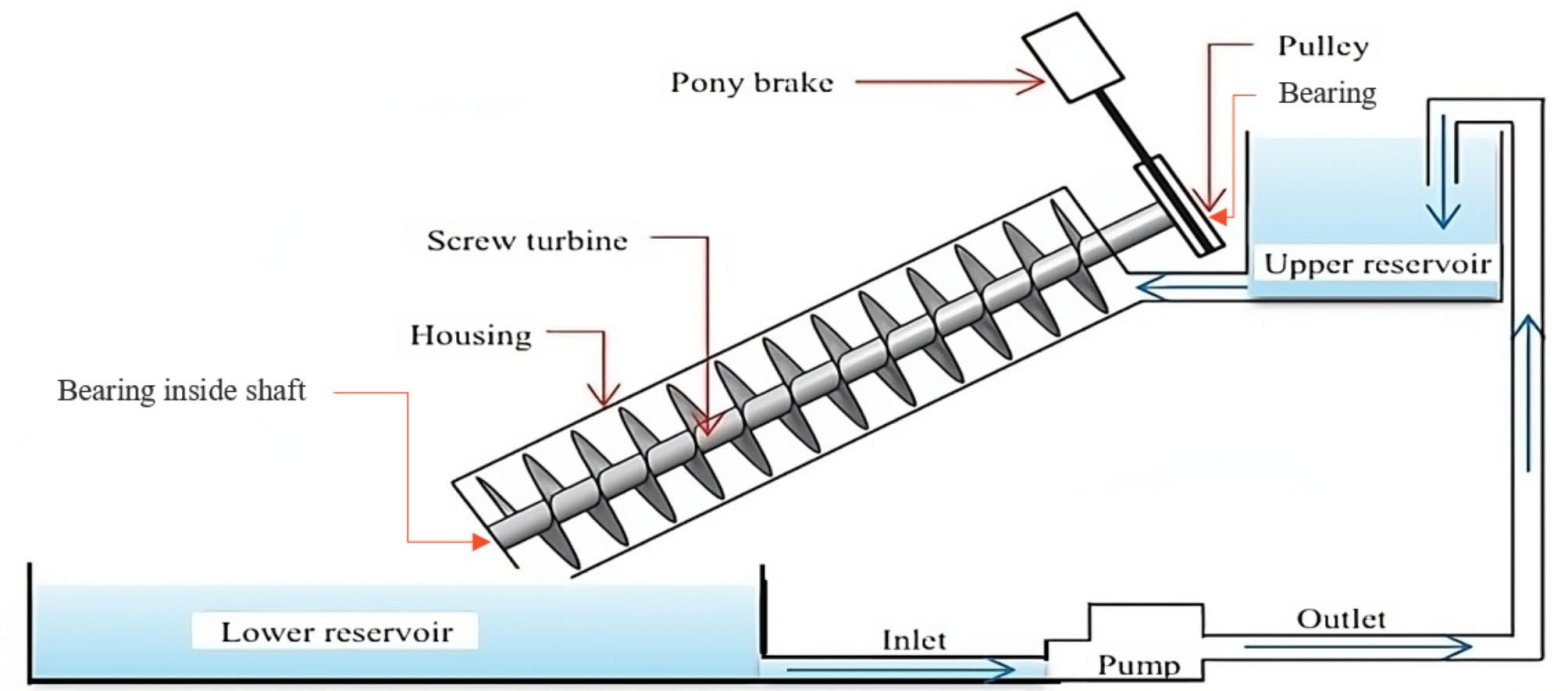
A lab setup of Archimedes turbine experimentation^33^

Previous studies are done by fixing installed (non-portable) AST having a chamber (tank) of water at a certain elevation to provide water to the turbine, as shown in Fig. 1. However, limited research has been conducted on portable-size Archimedes screw turbines^32^. This study aims to address this gap by analyzing the output power and overall efficiency of a portable Archimedes screw turbine at different flow rates and shaft inclination angles. This type of turbine does not include a water chamber, but the turbine metal plate in the waterway will block the flow of water, causing a rise in the water level head. A current study was conducted to design the geometric parameters of the Archimedes screw turbine. The key parameters included the length of the screw (L), number of blades (N), external diameter (De), internal diameter (Di), and blades geometry (α and β). After the designing of AST model was manufactured of stainless steel material and then tested at different flow rates and angle of inclination of screw shaft to analyze the power output and efficiencies. The turbine was locally manufactured in collaboration with a steelworks manufacturer, utilizing their workshop facilities and expertise in steel fabrication. As 3D printing presents a potential solution for addressing manufacturing imperfections in portable Archimedes screw turbines, its viability for large-scale production in low-resource areas must be carefully evaluated. Challenges such as material costs, production speed, and access to 3D printing technology may limit its practicality in remote areas. Additional research and testing are necessary to determine whether 3D printing can be effectively scaled for wider use in low resource areas. Computational analysis of this study on CFD will broaden the understanding and help to optimize Archimedes’ screw turbine.

## Methods and material

**Fig. 2:**
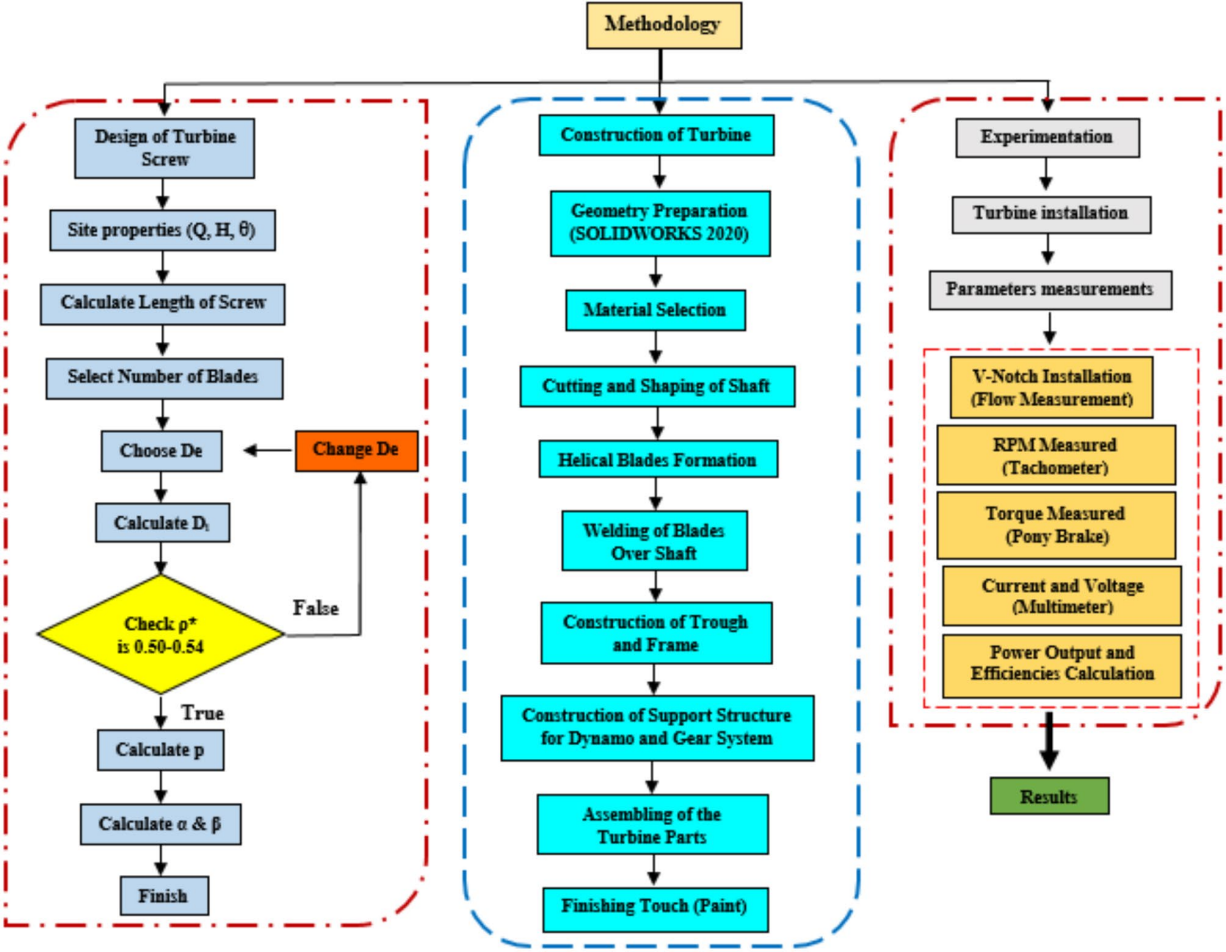
A detailed methodology flowchart for the AST study.

This section details the design, construction, and Testing of the turbine, along with the procedures and equipment used in the research work. The Fig. 2 methodology flowchart gives a general overview.

### Design of Archimedes screw turbine

The design of the AST is mainly concerned with the design of their Screw shaft.

### Design of screw

**Fig. 3:**
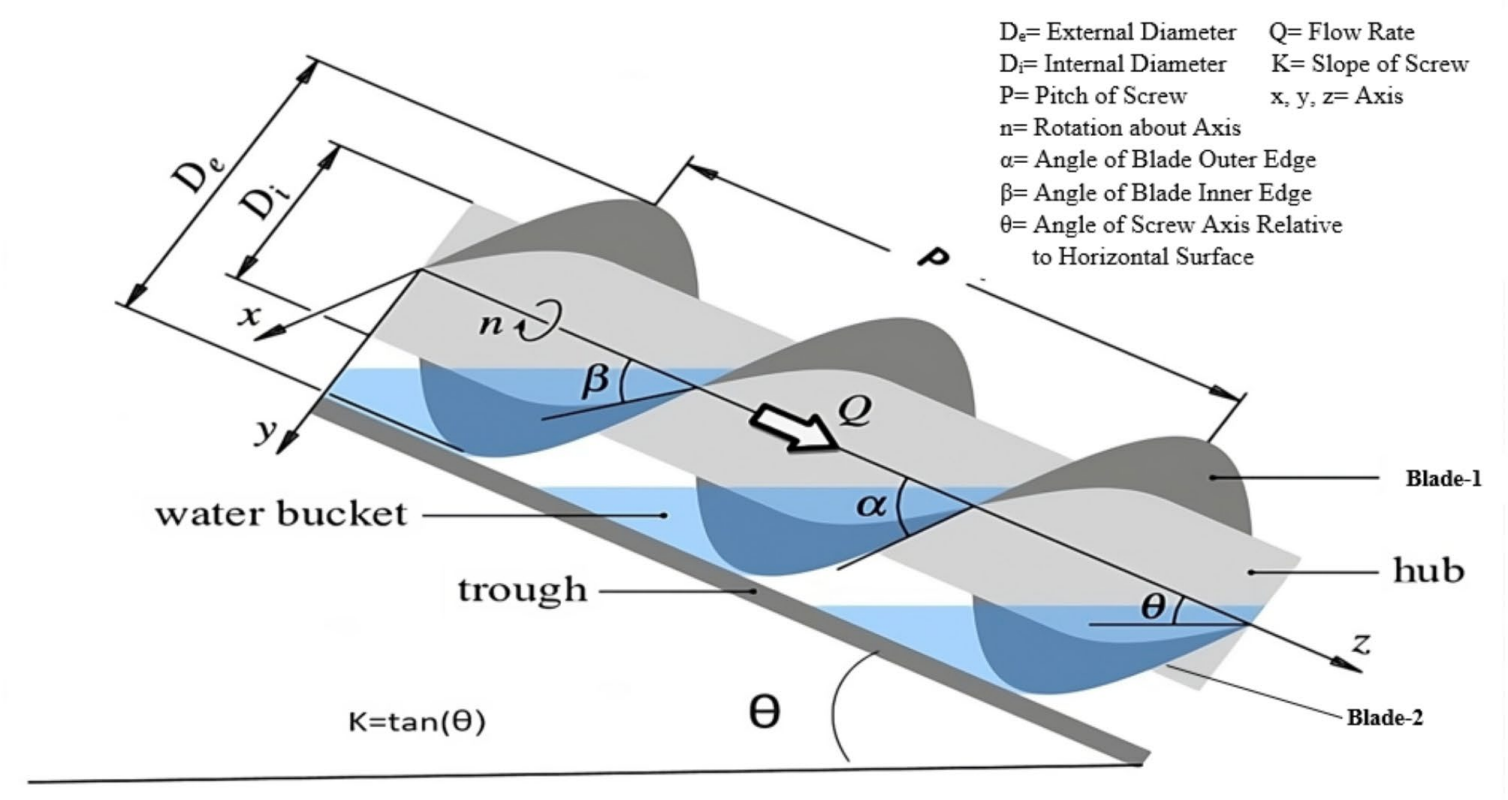
Profile view of turbine screw

The geometry of the turbine screw is governed by external parameters such as length (L), external diameter (De), and the angle of inclination (θ) of the screw, as well as internal parameters like the internal diameter of the screw (Di), the pitch of the blades (P), blade angles (α, β), and the number of blades (N). A schematic diagram, Fig. 3, represents these parameters of the screw. The values of these external parameters of the Archimedes screw depend on site conditions such as flow rate (Q), head (H), and the materials available for its construction^26^. The internal parameters of the screw depend on the external parameters.

#### Length of screw (L)

Generally, the length of the screw is selected according to passing flow rates (Q) and head of water (H) so that maximum available flows can be utilized. In the case of a Portable hydro-power turbine (PHPT), the length should be selected. Hence, the turbine is easily portable, or the following formula^11^ can also be used to find the length of the screw.

1 $$L = \frac{H}{{\sin \left( \theta \right)}}$$

where L is the length of the screw (cm), H is the head of water (cm), and θ is the angle of the screw shaft. Available head(H) and site slope θ is 22^0^. The length of the screw, according to Eq. (1), is 80 cm.

#### External diameter of screw (De)

The external diameter of an Archimedes Screw Hydro Turbine is important to its efficiency and power output. An increased external diameter enables the screw to collect more water, hence enhancing the turbine’s hydraulic efficiency and power production, particularly at low heads. A larger diameter offers an increased cross-sectional area for water flow, resulting in enhanced energy conversion. However, enlarging the diameter also increases the weight of the screw, which may impose greater impacts on the shaft and bearings, thus increasing frictional losses and mechanical wear with time^34^. The external diameter must be carefully determined according to site-specific factors, including water flow, head, and channel dimensions^35^. The turbine’s external diameter is selected at 18 cm according to the site channel available for the testing and flow rate available.

#### Angle of inclination (θ) of the screw

The inclination angle of the Archimedes screw depends on the slope of the installation site. However Lashofer et al.^36^ confirmed that many current industrial ASTs are installed at β = 22°. Inclination angles less than about 20° increase the length of the screw, and more than 30° lead to or considerably decrease the capacity of the screw^11^. In the current study, the inclination angle of the screw (θ) of the turbine model was considered 22° initially, which was then increased to 55° for testing purposes to analyze the turbine at different angles and flows.

#### Number of blades (N)

The main consideration for determining the number of blades in an Archimedes Screw Turbine (AST) was to optimize power delivery to the shaft while maintaining a lightweight runner. Augmenting the number of blades leads to a greater formation of water buckets between them, although concurrently diminishes the capacity of each bucket. Moreover, an enhanced number of blades may result in amplified shock losses at the runner’s entrance and enhanced viscous losses within the runner^34^. The inclusion of additional blades increases the weight of the screw, hence increasing the stress on the shaft and bearings, as well as the friction losses inside the bearings. Moreover, an excessive number of blades might diminish the effective volume of the runner.

Research by Lyons et al.^35^ indicates that expanding the number of blades beyond three (*N* = 3) yields only a negligible improvement in efficiency. Songin et al.^34^, noted that incorporating more than three blades does not significantly enhance the performance of a screw turbine. Consequently, it is typically concluded that the ideal number of blades for an AST is three.

Due to the manufacturing, weight and cost constraints, In modern screws, the optimal number of blades for an AST is taken as 1, 2 or 3^37^, for this research study screw of two blades was considered to utilize small flows effectively.

#### Internal diameter of screw (Di)

The internal diameter of the screw depends on the external diameter. Archimedean screws operating both as pumps and as turbines are consistent and suggest that a good trade-off would be a diameter ratio defined as the ratio of the inner diameter to the outer diameter of the screw and its range from 0.5 to 0.55. The ratio ρ = 0.54 found by Nuernbergk et al.^15^ as optimal for ASPs seems to be adequate for ASTs as well when the number of blades ranges from 1 to 5, as pointed out by Dragomirescu et al.^20^.

An optimal internal diameter of a screw was determined by Eq. 2 Rorres et al.^26^.

2 $$D_{i}^{*} = \rho ^{*} D_{e}$$

ρ^*^ is the optimum Diameter ratio, and De is the External diameter of the Screw. The value of ρ* is selected from Table 1. The internal diameter calculated using Eq. (2) was 9.6 cm. Table 1 gives the optimum ratio parameter.

**Table 1:** Optimum ratio parameters Rorres et al.^26^.

Number of screws	ρ^*^
1	0.5358
2	0.5369
3	0.5357
4	0.5353
5	0.5352

#### Pitch of the screw (p)

It is the distance between two adjacent points lying at the same height from the axis of the screw of the same blade, as shown in Fig. 3. For obtaining optimal performance, the blade pitch should be from 1.6 to 2.4 times higher than the outer radius Re and a standard value of p = De Nagel et al.^38^ should be adopted to avoid too many design variants and increased manufacturing costs. The pitch for this study was equal to D_e_, which was 18 cm.

#### Angles of the blade with shaft axis

The curvature of the blades is designed with angles, to hold and transport water effectively as it flows down the screw. Proper curvature ensures minimal water leakage or spillage between the flights (the individual spirals), maintaining high efficiency in energy conversion. These angles, as illustrated in Fig. 3, are α & β. The angle α is the angle formed by the sinusoidal curve defining the blade’s outer edge with the screw’s axis. Similarly, the angle β is the angle formed by the sinusoidal curve defining a blade’s inner edge with the screw’s axis. Equations (3) & (4) Dragomirescu et al.^20^ were used to find α & β, which were 72.30° and 58.90°, with the longitudinal axis of the screw shaft respectively.

3 $$\text{Tan} \alpha = \pi D_{e} {\text{/}}p$$

4 $$\text{Tan} \beta = \pi D_{i} {\text{/}}p$$

where D_e_ and D_i_ are the external and internal diameter of the screw, and p is the pitch of the screw blades.

### Design parameters of turbine screw

Table 2 provides the design parameters of the studied screw.

**Table 2:** Design parameters of the studied screw.

Designing parameters	Values	Units
External diameter (D_e_)	18	cm
Length of screw (L)	80	cm
Slope of screw (K)	22°–55°	Degree
Internal diameter (Di)	9.6	cm
Pitch of the screw (p)	18	cm
Angles of the blade (α,β)	α = 72.3°, β = 58.9°	Degree
Number of blades (N)	2	No.

### Manufacturing of the Archimedes turbine model

**Fig. 4:**
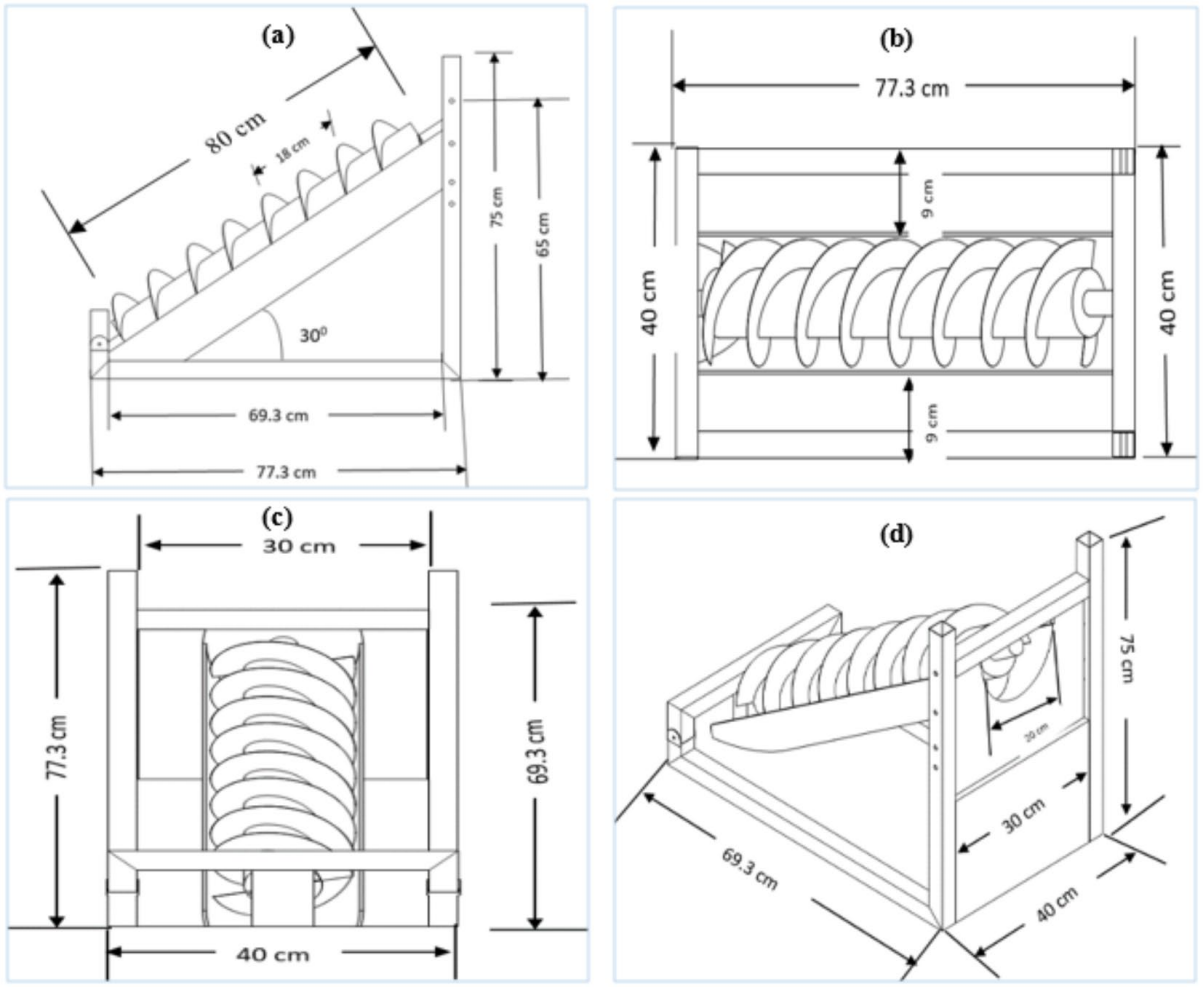
(**a**) Side view of the turbine, (**b**) top view of the turbine, (**c**) front view of turbine and (**d**) isometric view of turbine

**Fig. 5:**
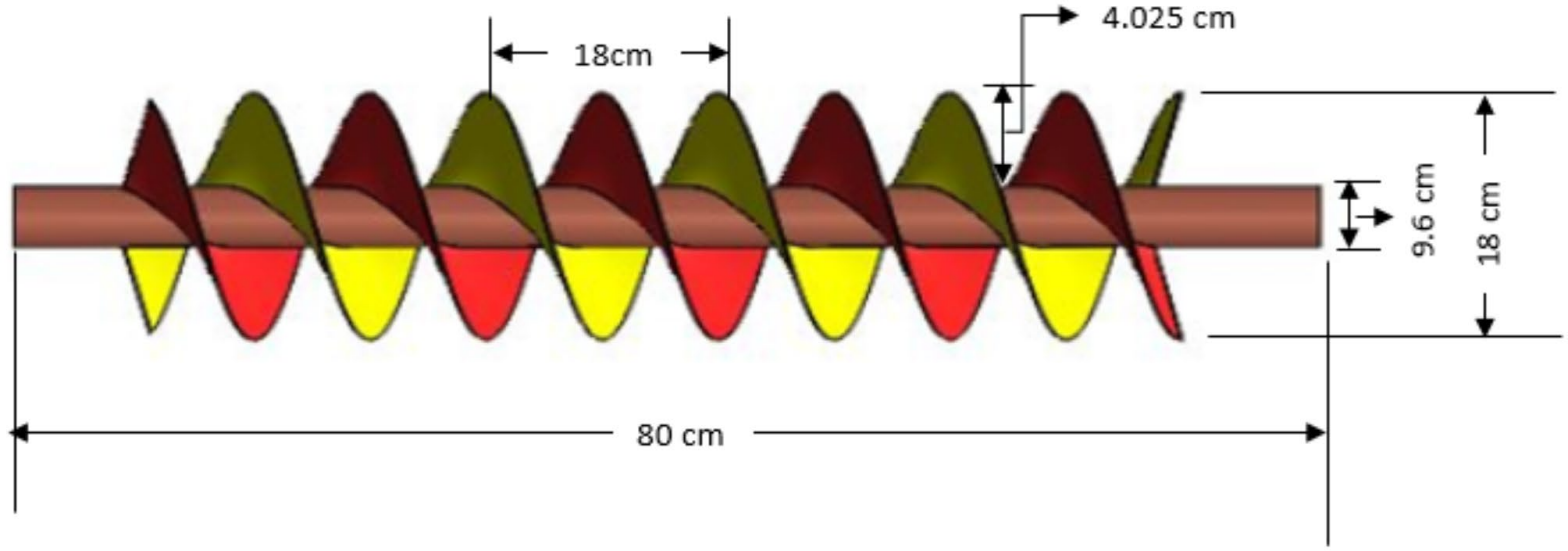
Geometrical parameters of screw shaft

**Fig. 6:**
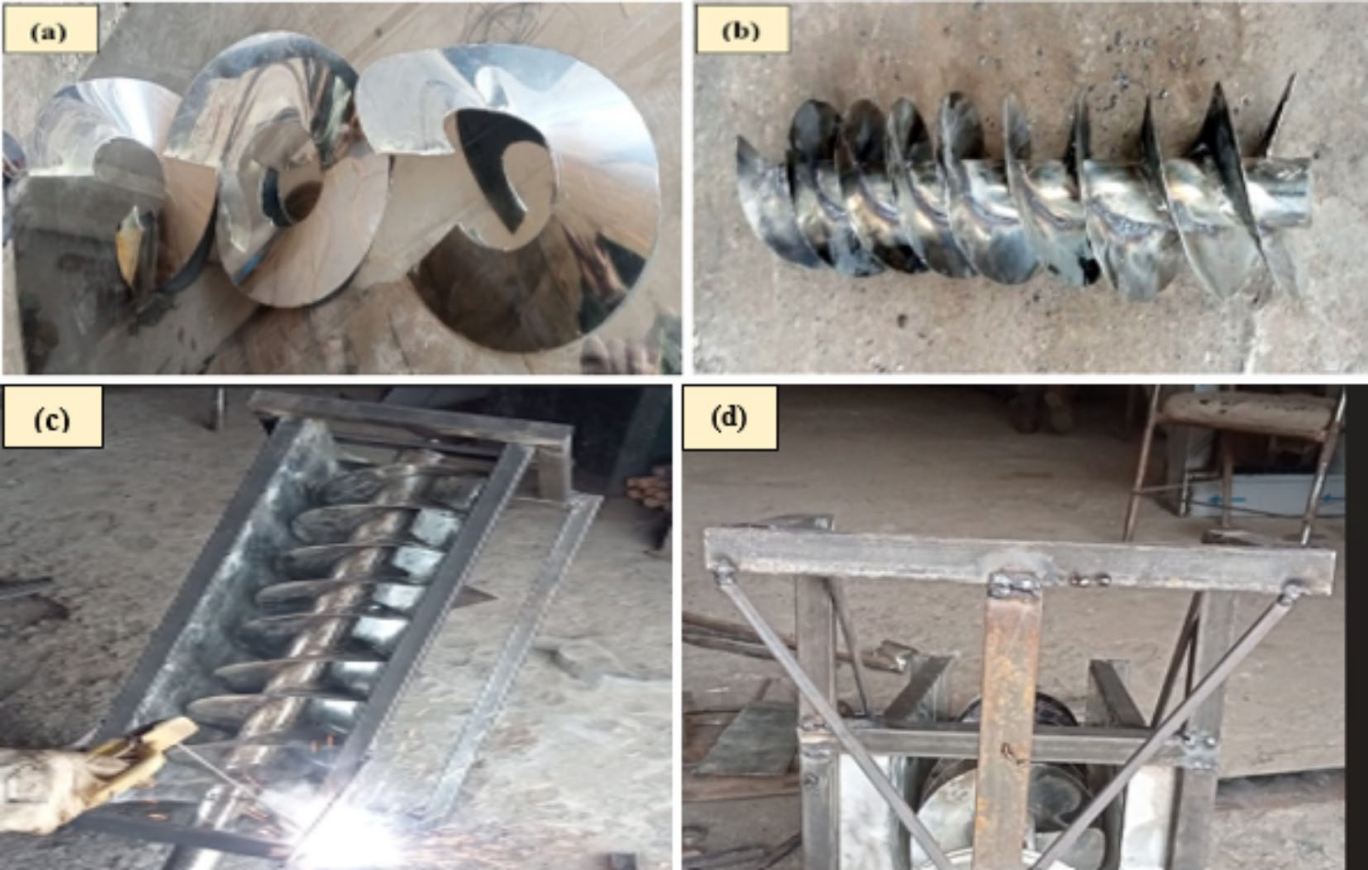
(**a**) Helical blades, (**b**) blades wrapped around the shaft, (**c**) manufacturing of trough and frame, and (**d**) support structure for Dynamo and Gear-system.

**Fig. 7:**
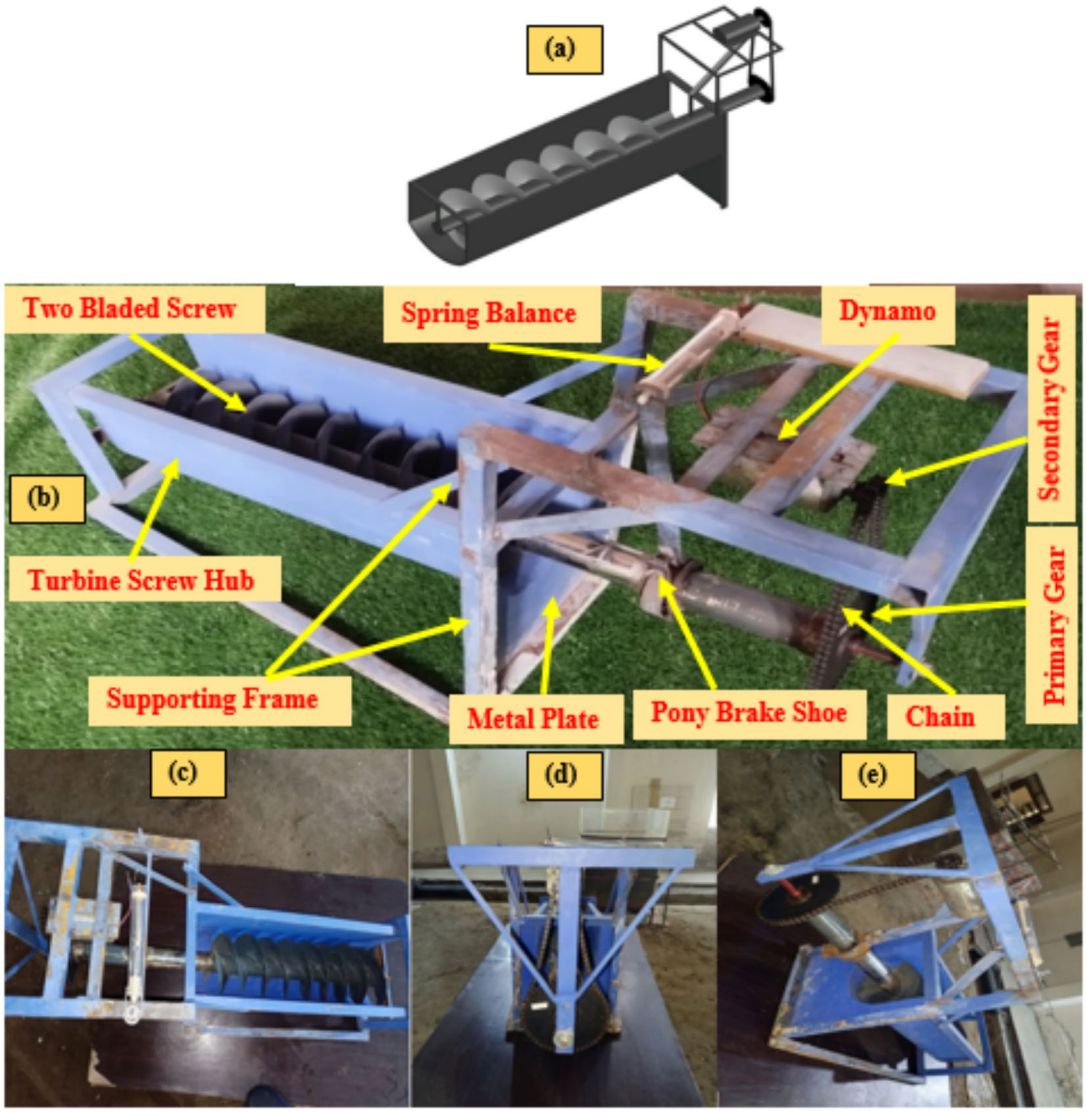
(**a**) A 3D representation of turbine model in SOLIDWORKS, (**b**) complete detail of AST model with labels and representation of orthographic views, (**c**) top view, (**d**) front view, (**e**) side view.

Design drawings of the Archimedes Screw were created in SOLIDWORKS 2020. The detailed design diagrams, complete with dimensions, are presented in Figs. 4 and 5. Stainless steel material was used in the manufacturing. The process began with cutting and shaping the shaft. A stainless steel pipe was measured and cut to the required length using an electric grinder. For the helical blades of the screw shaft, a stainless-steel sheet was marked and cut according to the screw blade dimensions using an electric grinder and shaped into curved blades after heating, as shown in Fig. 6. These blades were then welded onto a hollow stainless steel shaft at regular intervals for even spacing and secure attachment, as shown in Fig. 7.

The turbine trough (web) and body frame were manufactured from U-shaped steel plates, providing housing for the screw. Braces were added to maintain stability and alignment, and the screw was attached to the frame using brackets (Fig. 6). Proper angling and leveling ensured efficient water flow.

For power transmission, a chain-drive system with a 1:12 gear ratio was installed to transfer the mechanical power from the turbine to the dynamo. The primary gear was fixed to the shaft, and bearings were inserted at both ends, securing the runner in the frame. A small structure was added to support and connect the AST shaft and dynamo through chain-driver system as illustrated in Fig. 6.

Finally, weld splatter and sharp edges were removed from the surface, and a protective paint coating was applied to prevent corrosion.

### Isometric and orthographic views of AST

Figure 7 provides a detailed labeled 3D representation of the AST along with orthographic views (top, front, side) to ensure a clear understanding of the turbine model shape and size.

## Experimentation

**Fig. 8:**
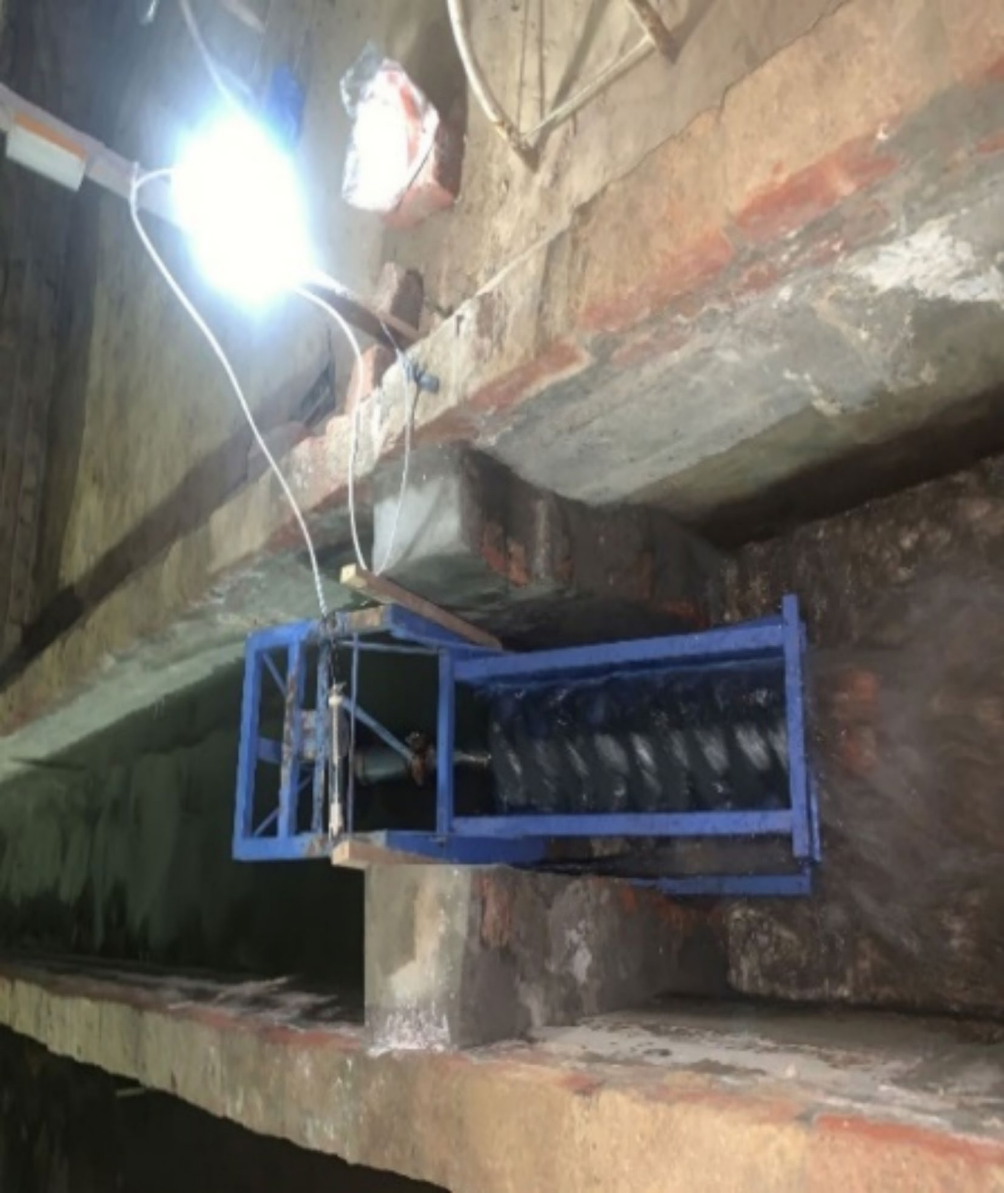
Installation of AST model in rectangular channel during experimentation.

There is no special installation required for the experimentation. The turbine model was placed in an open channel of water in the lab (Fig. 8). The width of the channel was adjusted according to the width of the model to ensure the desired flow of water. The model was tested at different angles of inclination of the shaft screw and flow rates. The adjustment of the model at different angles was done through the adjustable frame of the turbine.

### Measurement of flow parameters

To measure the flow rate, a V-notch was installed on the upstream side of the turbine. A gauge well was installed on the right side of the channel to measure the water head (H) relative to the bottom of the V-notch. The gauge well is a transparent vessel made of Plexiglas, with a measurement scale attached to its surface. The measured water level was then used in the discharge formula for the V-notch to calculate the discharge. Equation 5 was applied to determine the discharge through a 45° V-notch.

5 $$Q = 2.47~H^{{2.5}}$$

**Fig. 9:**
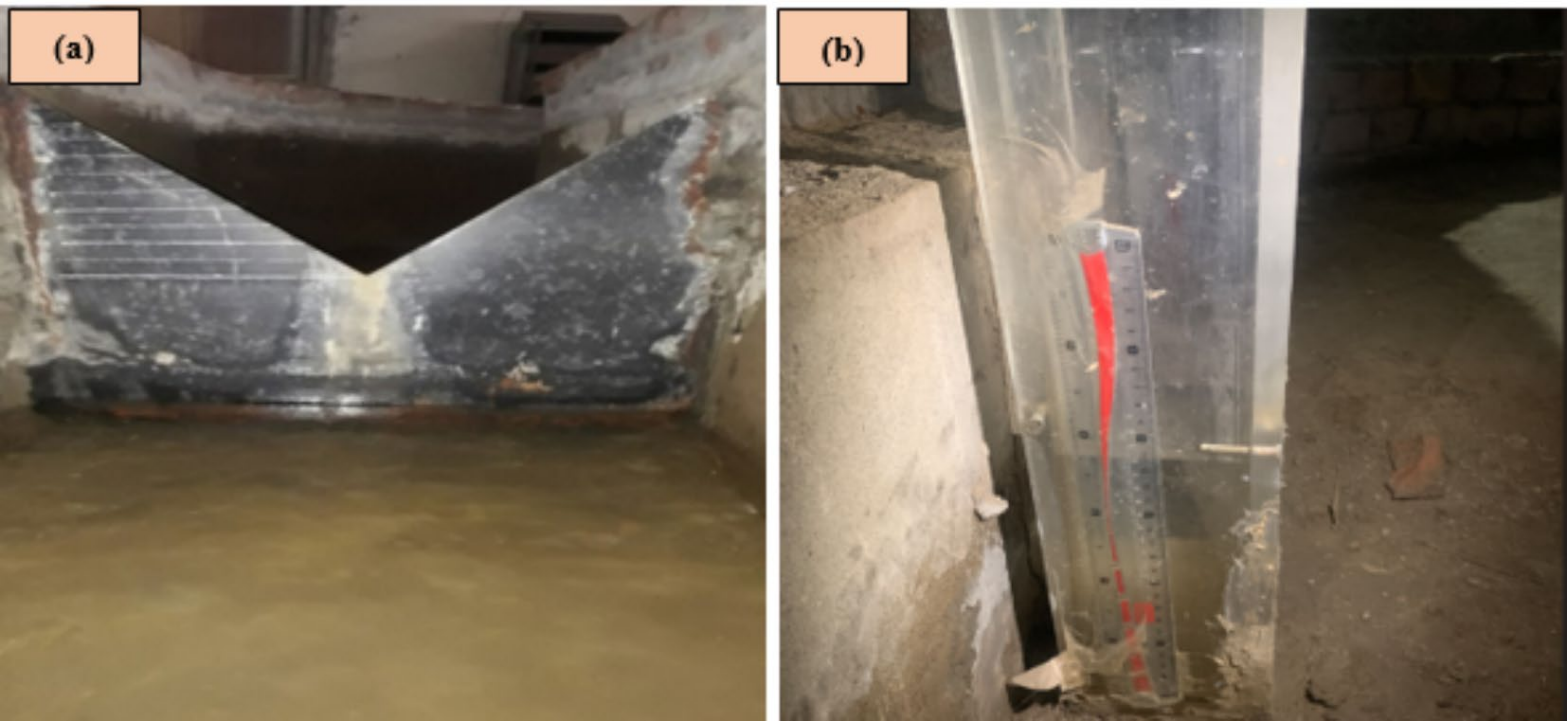
(**a**) 45° V-Notch for discharge measurement and (**b**) stilling well for measuring.

In the above equation, Q is discharge in ft^3^/sec, and H is the water head in ft upstream of the V-Notch. Figure 9a,b shows the V-Notch and gauge well respectively.

the water head above v-notch.

### Measurement of torque, RPM, current, and voltage

**Fig. 10:**
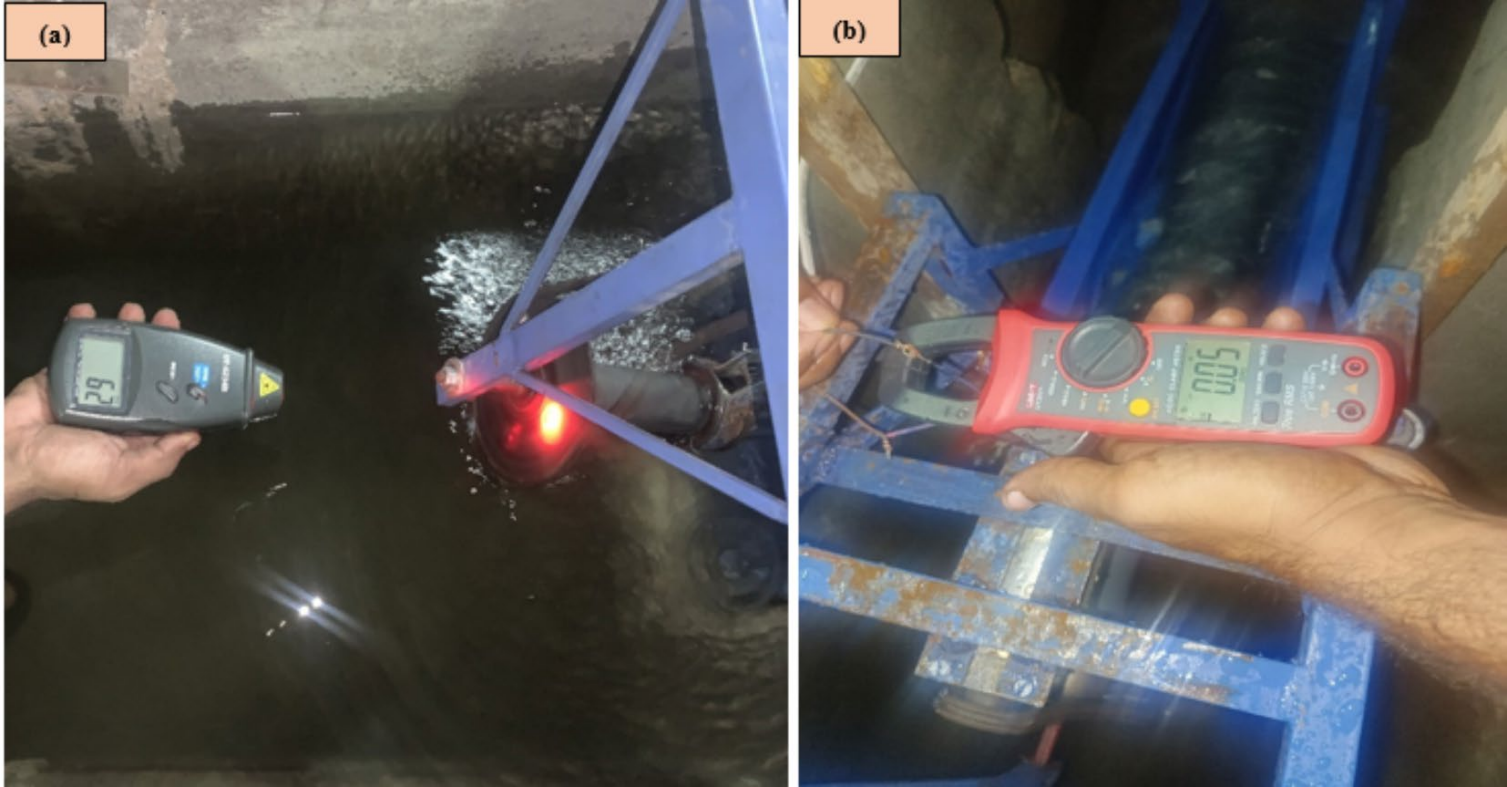
(**a**) Tachometer for measuring RPM and (**b**) Clamp meter for measuring voltage and current.

Torque was measured using a pony brake, where a brake shoe was attached to a lever arm. The brake shoe creates friction by pressing against the rotating shaft. The lever arm was connected to either a spring or a weight scale to balance the forces generated. As the shaft rotates, the friction between the brake shoe and the shaft produces a resisting force. The spring balance measures the resisting force. This resisting force is directly proportional to the torque generated by the shaft. Torque (T) is calculated using the Eq. 6:

6 $$T = F*r$$

where T is the torque (Nm) produced by the shaft, F is the force (N) measured at the end of the lever arm, and r is the length (m) of the lever arm (distance from the center of the shaft to the point where force is applied). Revolutions per minute (RPM) of the turbine shaft were determined using a digital tachometer, while electric current and voltage were measured using a multimeter. Figure 10 represents the tachometer for measuring RPM. Table 3 gives the values of RPM and other factors.

**Table 3:** Values of RPM, frictional force, Torque and Power output.

Sr. no	Flow Rate	Inclination Angle	No. of Revolution	Force	Torque	Power
	(ft^3^/s)	(Degree)	(RPM)	(N)	(Nm)	(Watt)
1	0.3	22	58	8.5	2.6	15.5
2	0.3	30	70	9.7	2.9	21.3
3	0.3	45	73	9.7	2.9	22.2
4	0.3	55	75	9.7	2.9	22.8
5	0.4	22	71	9.7	2.9	21.6
6	0.4	30	86	11.1	3.3	30
7	0.4	45	92	10.7	3.2	30.8
8	0.4	55	95	10.8	3.2	32.1
9	0.5	22	78	11.5	3.5	28.2
10	0.5	30	104	12.9	3.9	42
11	0.5	45	108	13.1	3.9	44.5
12	0.5	55	111	13.8	4.1	48.2
13	0.6	22	82	11.8	3.5	30.4
14	0.6	30	107	12.5	3.8	42
15	0.6	45	115	12.8	3.8	46.2
16	0.6	55	117	13	3.9	47.8
17	0.7	22	89	11.9	3.6	33.1
18	0.7	30	112	12.8	3.8	45
19	0.7	45	115	13.1	3.9	47.3
20	0.7	55	121	13.5	4.1	51.3

### Efficiencies and power calculations

Turbine power was obtained by multiplying torque with angular velocity using Eq. 7.

7 $$P_{{Shaft}} = \omega *T$$

where ω (rad/s) is the angular velocity of the shaft, and T is the torque (Nm) produced by the shaft. The angular velocity (ω) is obtained from the equation ω = 2 π n /60, and n is the rotational speed (rpm). Hydraulic power was calculated using Eq. 8.

8 $$P_{{hyd}} = \rho ghQ$$

where $\:\rho\:$ is the density of water (kg/m^3^),$\:\:g$ is the gravitational acceleration (m/s^2^), ℎ is the head of water (m), and Q is the flow rate of water (ft^3^/s).

Hydraulic Efficiency, Power from dynamo, mechanical efficiency and overall efficiency were computed using Eq. 9 to 12 respectively.

9 $$\eta _{{hyd}} = P_{{Shaft}} /P_{{hyd}}$$

10 $$P_{{dynamo}} = v*l$$

where v is voltage, and l is current produced from the dynamo.

11 $$\eta _{{mechanical}} = P_{{Shaft}} /P_{{dynamo}}$$

12 $$\eta _{{overall}} = \eta _{{hyd}} *\eta _{{mechanical}}$$

## Results and discussion

This study investigated the performance of a turbine model under various operating conditions, testing four inclination angles (22°, 30°, 45°, and 55°) and five flow rates (0.3, 0.4, 0.5, 0.6, and 0.7 ft^3^/s). Results regarding hydraulic power, mechanical power, hydraulic efficiency, mechanical efficiency and overall efficiency of the turbine are listed in Table 4.

**Table 4:** Efficiency calculations at different flow rates and inclination angles.

Sr. no	Flow rate	Inclination angle	No. of revolution	Hydraulic power	Mechanical power	Hydraulic efficiency	Mechanical efficiency	Overall efficiency
	(ft^3^/s)	(Degree)	RPM	(Watt)	(Watt)	(%)		(%)
1	0.3	22	58	25	15.5	62.16	0.92	57.19
2	0.3	30	70	33.4	21.3	63.61	0.92	58.53
3	0.3	45	77	46.7	22.2	48.32	0.92	44.46
4	0.3	55	79	54.2	22.8	42.87	0.92	39.44
5	0.4	22	71	33.3	21.6	64.72	0.92	59.54
6	0.4	30	86	44.3	30	67.66	0.92	62.25
7	0.4	45	92	62.1	30.8	49.62	0.92	45.65
8	0.4	55	95	72.1	32.1	44.55	0.92	40.98
9	0.5	22	78	41.8	28.2	67.38	0.92	62
10	0.5	30	104	55.7	42	75.55	0.92	70
11	0.5	45	108	78	44.5	57.1	0.92	52.53
12	0.5	55	111	90.5	48.2	53.22	0.92	48.97
13	0.6	22	82	50	30.4	59.91	0.92	55.11
14	0.6	30	107	66.7	42	64.14	0.92	59.01
15	0.6	45	110	93.4	46.2	47.34	0.92	43.55
16	0.6	55	113	108.4	47.8	42.66	0.92	39.25
17	0.7	22	87	58.3	38.1	54.82	0.92	50.44
18	0.7	30	114	77.7	46.3	59.58	0.92	54.82
19	0.7	45	122	108.8	50.1	46.01	0.92	42.33
20	0.7	55	135	126.3	55.7	44.08	0.92	40.55

**Fig. 11:**
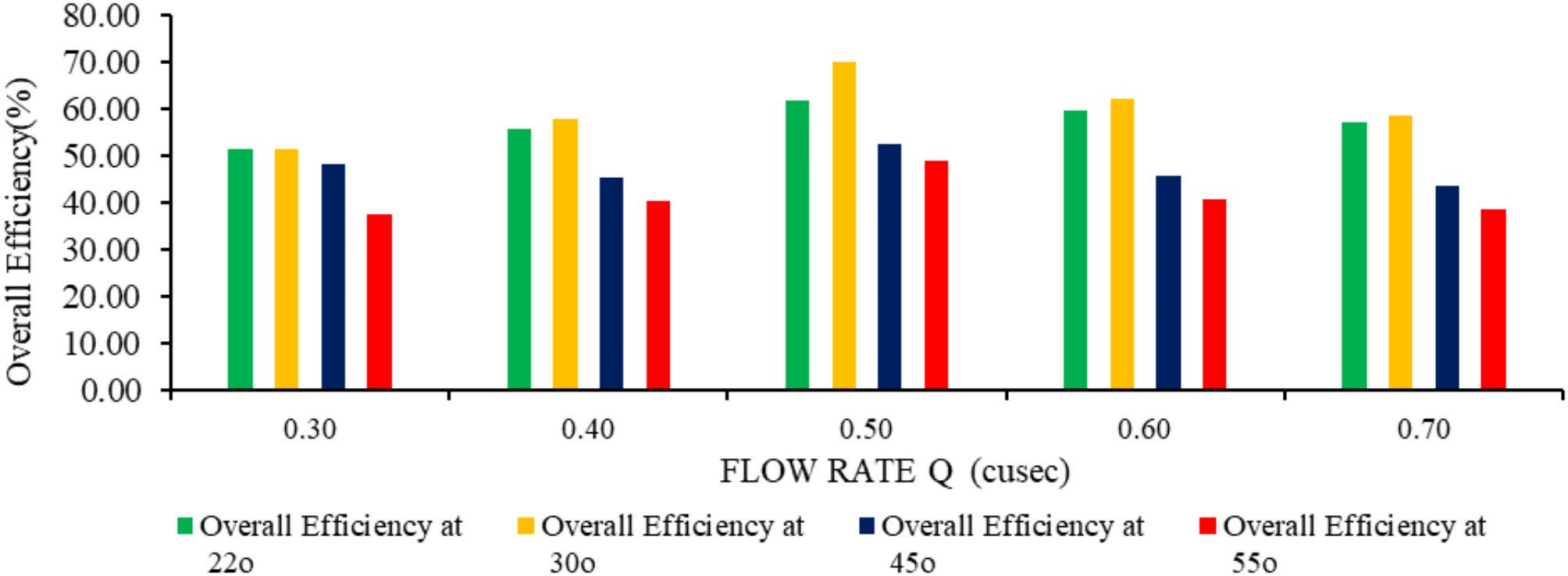
Flow rate and overall efficiency at different angles of inclination.

Table 4 indicates that overall efficiency is in direct relation to hydraulic efficiency. The highest overall efficiency achieved was 70% against hydraulic efficiency of 75.5% at 30° of angle of inclination at a flow rate of 0.5 ft^3^/s. An increase in the angle of inclination above 30° started increasing the water losses. Angle of inclination greater than 30° started pushing water directly outwards from the trough of the turbine. When the inclination angle surpassed its optimum point, the direction of the water weight force would likely be more parallel to the direction of the normal vector of the gap surface between the blade and the trough^39^. Therefore, most of the water weight force pushed the water through the gap leakage, which resulted in a large gap flow leakage, and lower overall efficiency. A study Lashofer et al.^36^ analyzed 74 Archimedes Screw Turbines (ASTs) at 71 European sites, revealing an average overall efficiency of 69%. Most ASTs have been installed at an inclination angle of 22. Several studies have also suggested that the optimum inclination angle of an AST should be less than 25°^40^. some studies have fined The optimum sc^o 36^rew turbine shaft slope greater than 30°^41,42,43^. These different optimum inclination angles from several studies were certainly influenced by other geometric parameters, such as the number of blades *N* and the pitch^44^. It was mentioned Shahverdi et al.^45^ that the overall efficiency of the AST decreases when the inclination angle exceeds 30°. Figure 11 represents flow rate and overall efficiency.

**Fig. 12:**
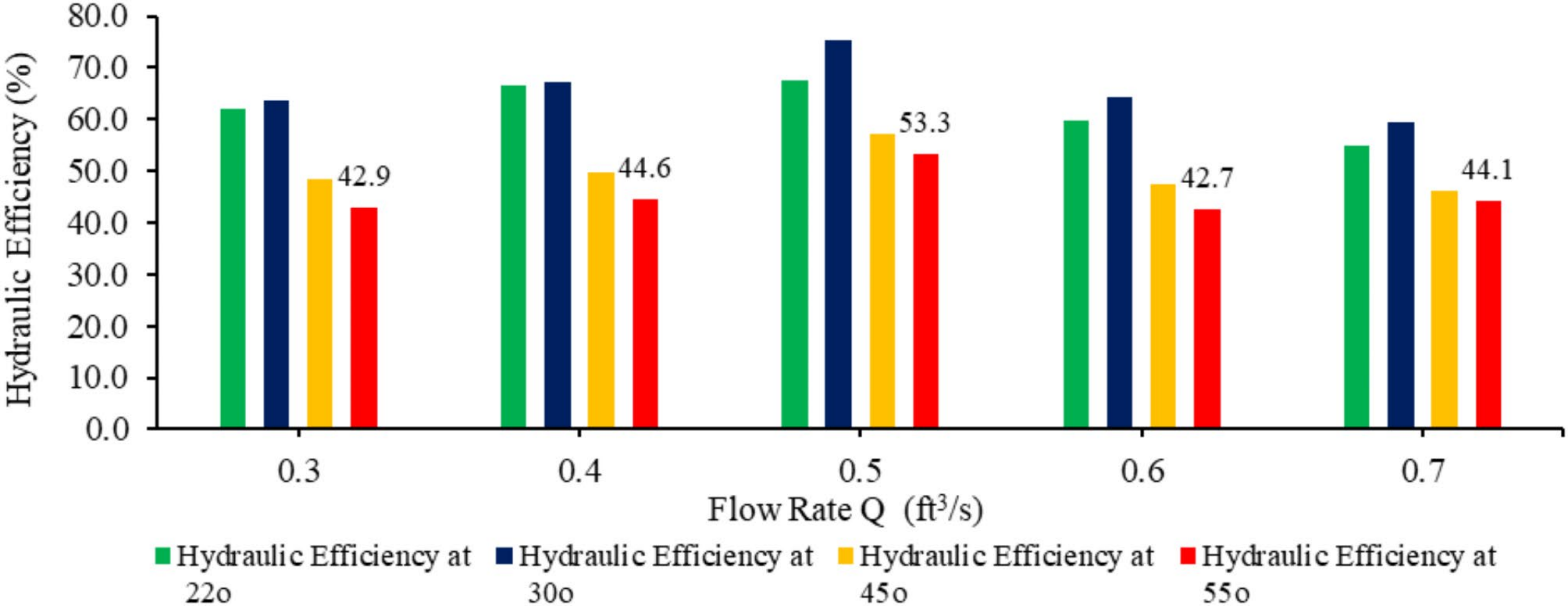
Relation between flow rate and hydraulic efficiency with respect to angle of inclination.

At 22 and 30-degree inclinations, the hydraulic efficiency for all flows (0.3,0.4,0.5,0.6 and 0.7) increases as shown in Fig. 12. For each flow rate their peak hydraulic efficiency is obtained at 30° angle of inclination. Overall the maximum hydraulic efficiency of 75.5% is obtained at 0.5 ft^3^/s .This suggests that the optimal operating conditions for the turbine lie around a flow rate of 0.5 ft^3^/s and at a 30-degree inclination. The total flow rate that enters the turbine could be divided into 3 elements, i.e., effective flow, Q_effective_, gap flow leakage (Q_gap)_, and overflow leakage (Q_overflow)_^15^. Q_effective_ is the effective flow rate that contributes to driving the turbine, while both Q_gap_ and Q_overflow_ are leakage flows that can reduce the turbine’s efficiency. Q_g_ is the leakage flow due to the gap between the blade and the trough, and Q_overflow_ is the leakage flow that occurs when the turbine is overfilled, and the water level inside the turbine exceeds its optimum filling level.Above 30° inclination of screw shaft and flows of 0.3 ft^3^/s and 0.4 ft^3^/s the buckets of screw is not optimally filled results in low hydraulic efficiency.The decline in hydraulic efficiency at higher flows of 0.6 ft^3^/s and 0.7 ft^3^/s is due to overflow losses. as shown in Fig. 12.

**Fig. 13:**
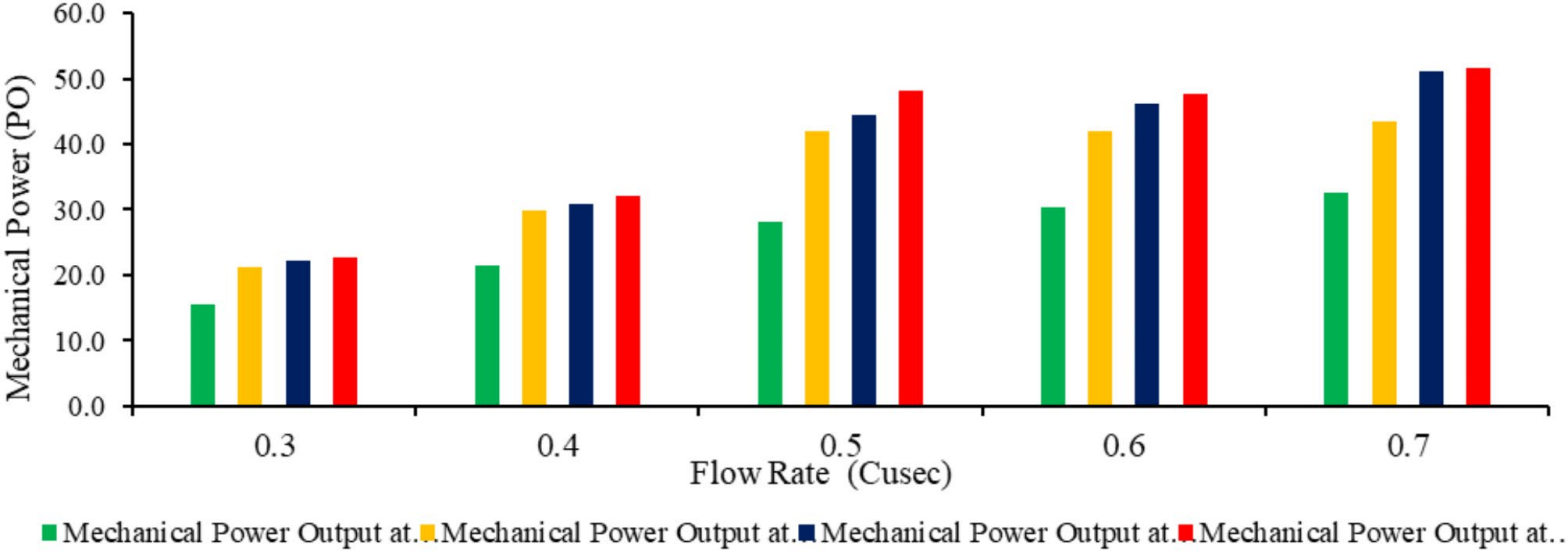
Relation between flow rate and mechanical power output with respect to angle of inclination.

As the flow rate increases, Mechanical power output raised across all inclinations, with the 55° inclination consistently achieved the highest output, peaking at 55.7 W at 0.70 ft^3^/s. The 30^o^ and 45^o^ inclinations also perform well, particularly at higher flow rates, reaching 46.5 and 50.3 watts, respectively, at 0.70 ft^3^/s. In contrast, the 22° inclination delivered the lowest mechanical power output across all flow rates, indicating it was the least efficient. Overall, the 55° inclination was the most effective for maximizing mechanical power, particularly at higher flow rates. However, the overall efficiency was maximum at 0.5 ft^3^/s flow at 30° of inclination angle. The turbine’s mechanical efficiency was 92%. Rohmer et al.^46^ investigated the performance of Archimedes screw turbines because of the effect of flow rates on torque, power and efficiency. The results of this study indicate that to obtain maximum mechanical power, flow conditions must be maintained at the highest water flow rate conditions, even though the efficiency obtained is not the maximum value. Figure 13 shows relation between flow rate and mechanical power.

**Fig. 14:**
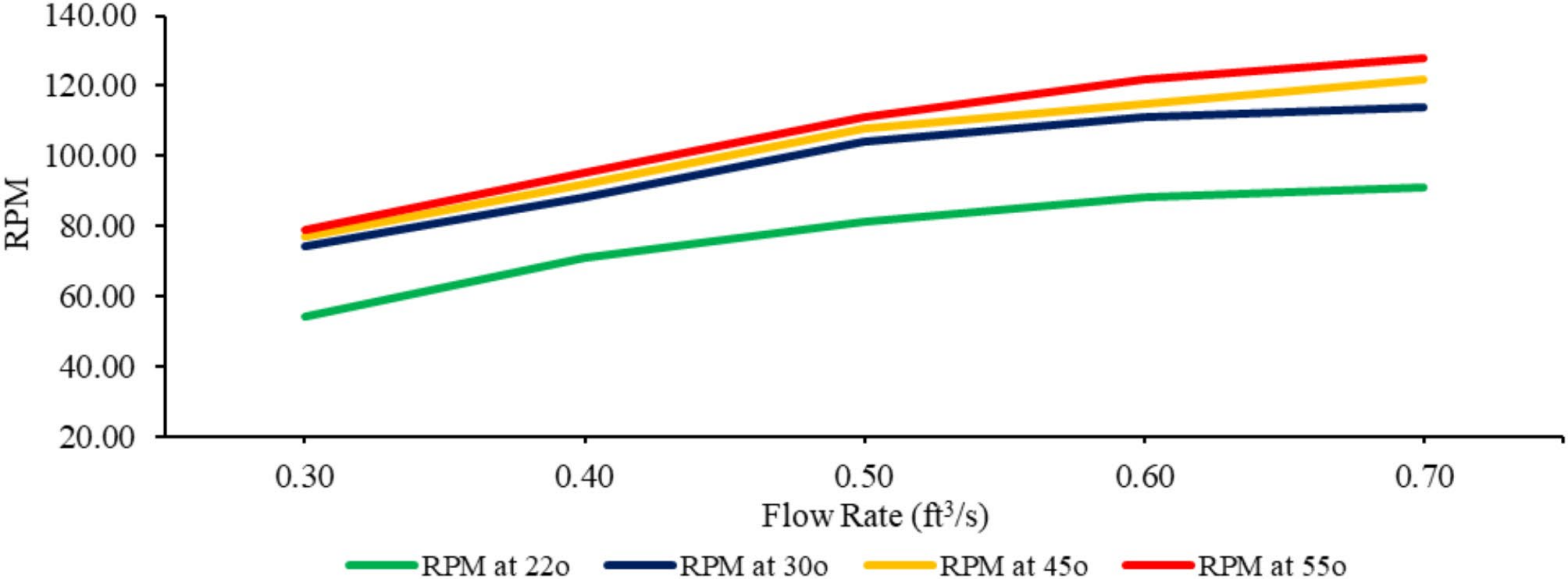
Flow rate and RPM at a different angle of inclination.

Revolution per minute (RPM) is directly measured output. An increase in flow rate is gradually increasing the RPM of the turbine^47^. At the lowest angle of inclination, i.e., 22°, it has been observed that the increase in RPM between the trends of 22° and 30° is not gradual, as shown in Fig. 14. This abrupt increase in RPM is because more compressed water is hitting the blades of the screw. When the inclination angle of the AST exceeds 30°, the buckets of the screw are not filled with water. This results in a phenomenon where the water tends to spill from the buckets during the rotation of the screw, leading to water losses. However, the RPM is still increasing gradually due to an increase in the volume and head (H) of water, as shown in Fig. 14. Due to more water losses, the hydraulic efficiency decreases, which adversely impacts the overall efficiency of the turbine. Maulana et al.^30^ determined that besides the increase in RPM, it contributes to the increase of mechanical power. Still, overall efficiency is decreased due to the inability to harness the volume of water passing through the turbine significantly.

**Fig. 15:**
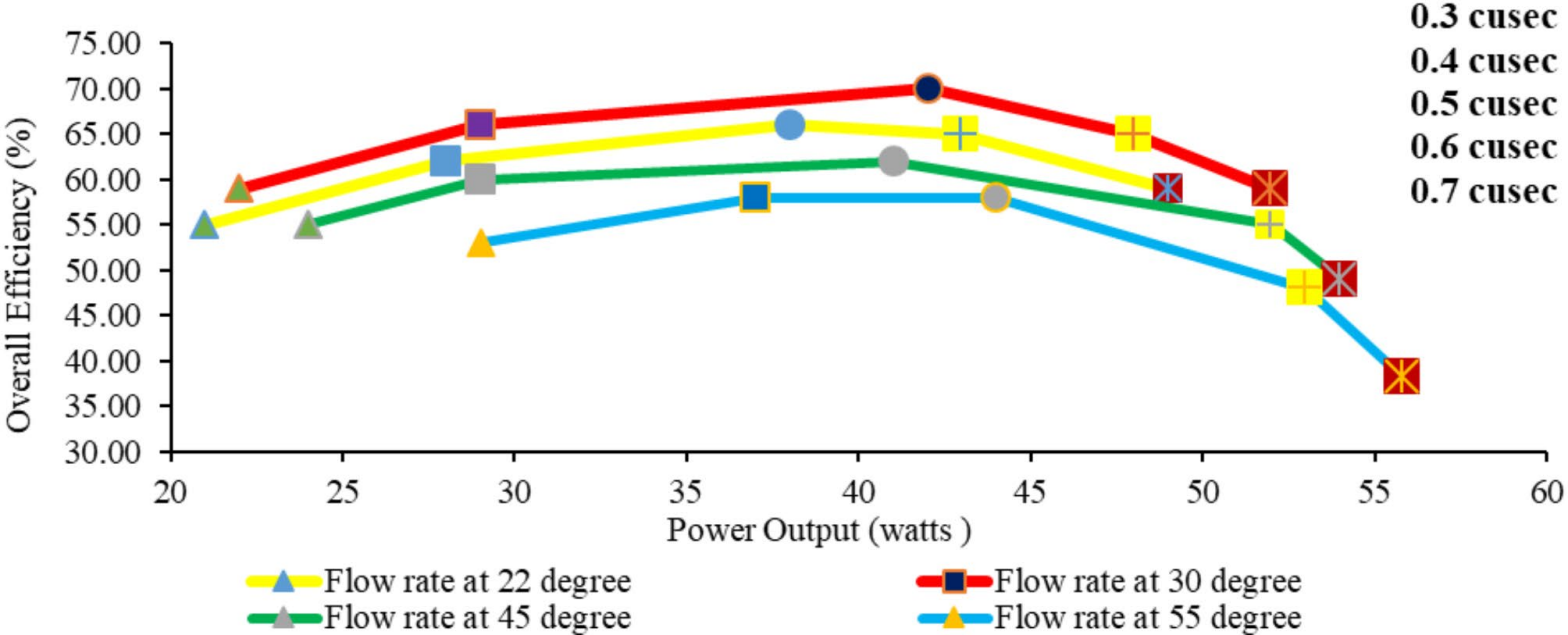
Relation between overall efficiency and power output with respect to angle of inclinations at different flows.

Figure 15 indicates that the optimal combination of flow rate and angle of inclination for achieving high overall efficiency lies at moderate angles (30°) and moderate flow rates (around 0.4 to 0.5 ft^3^/s). At higher flow rates (0.6 and 0.7 cusec) and angles above 30°, the system experiences diminished overall efficiency despite the increase in power output due to mechanical and fluid dynamics limitations (turbine capacity and hydraulic losses) at steeper angles and larger flow volumes. Hydraulic losses significantly decrease hydraulic efficiency, which results in a decrease in the overall efficiency of the AST, as overall efficiency is the combination of hydraulic and mechanical efficiency. Rohmer et al.^48^ find that the turbines with high power conditions (i.e., flow rates) do not necessarily have high efficiency because several factors affect the performance, such as hydraulic losses.

## Conclusions

An Archimedes screw turbine was designed on the basis of geometric parameters of the turbine screw shaft. The geometry of the turbine screw consists of external parameters such as length (L), external diameter (De), and the angle of inclination (θ) of the screw, and internal parameters like the internal diameter of the screw (Di), the pitch of the blades (P), blade angles (α, β), and the number of blades (N). On designing, drawings of turbine model were prepared in Solidworks. The physical model of the turbine was manufactured of stainless steel material as per design. After manufacturing, experimentation was carried out to evaluate turbine power output and efficiencies at different operating conditions.

Turbine was tested at various discharges of 0.3, 0.4, 0.5, 0.6 and 0.7 ft^3^/s and shaft angles of 22°, 30°, 45° and 55°. The optimum turbine power output (P_o_) of 42 watts and overall efficiency (η_o_) of 70% were achieved at a flow rate (Q) of 0.5 ft^3^/s and an angle of inclination of the shaft slope of 30°. With an hydraulic efficiency of 75.5% in this case. The highest Power output (P_o_) from the turbine was 55.7 watts at a flow rate of 0.7 ft^3^/s and shaft slope of 55°. However, there was no optimum Power output (Po) because of the low hydraulic efficiency (44%) of the turbine at these operating conditions, which resulted in a decrease in the overall efficiency (41%) of the turbine. The results of the testing showed that the flow rate (Q) and inclination of the turbine shaft affect the turbine power output (P_o_) and overall efficiency (η_o_).

The portable (AST) offers substantial scalability potential, particularly in off-grid rural communities. The compact and transportable nature of the turbine allows for easy deployment in various environments, providing a versatile solution for sustainable energy generation in diverse settings. Its ability to operate in low-flow conditions further enhances its scalability, making it suitable for a variety of water sources, including small rivers, canals, and irrigation channels, which may not be suitable for traditional hydroelectric turbines. ASTs require regular maintenance to address wear, corrosion, and sediment buildup, especially in remote or harsh environments. The Logistical challenges include transportation to remote areas with limited resources, while environmental conditions such as water flow variability and ecosystem impact must be carefully considered during deployment.

Although 3D printing offers a promising solution to address manufacturing imperfections in the development of portable Archimedes screw turbines, its feasibility for large-scale production, particularly in low-resource areas, requires careful consideration. Factors such as material costs, production speed, and the availability of 3D printing technology may limit its practicality in such contexts. Further research and experimentation are needed to assess whether 3D printing can be effectively scaled for broader implementation in these low-resource areas.

Future researchers will use the data from these tests to provide a thorough computational examination of the AST model using CFD simulations. This method will provide a thorough comparison of physical and numerical model outcomes, permitting the examination of total losses, efficiency, and power production across different flow rates and angles of inclination. The use of CFD analysis will enhance turbine design optimization and refine performance predictions.

## Data Availability

The datasets used and/or analyzed during the current study are available from the corresponding author upon reasonable request.
